# High-throughput *in silico* multi-epitope reverse vaccine design against hydatidosis

**DOI:** 10.1038/s41598-026-50570-7

**Published:** 2026-08-11

**Authors:** Dania Skhal, Samar Al Nahhas, Osama Skhal, Abdul Qader Abbady

**Affiliations:** 1 Department of Animal Biology, Faculty of Science, Damascus University, Damascus, Syria; 2 Ministry of Interior, Damascus, Syria; 3 Division of Molecular Biomedicine, Department of Molecular Biology and Biotechnology, Atomic Energy Commission of Syria (AECS), Damascus, Syria

**Keywords:** *Echinococcus granulosus*, Immunoinformatic, Multi-epitope vaccines, Epitopes prediction, *In silico* cloning, Biotechnology, Computational biology and bioinformatics, Immunology, Microbiology

## Abstract

The Middle East is a significant hotspot for human hydatidosis caused by *Echinococcus granulosus*, a complex multi-stage pathogen exhibiting antigenic variation with diverse epitopes. Multi-epitope vaccines, designed using immunoinformatics, represent a promising approach to effectively control this challenging parasite. A total of 1150 secreted, non-toxic, antigenic proteins were obtained from database and analyzed for their immunogenic potential, leading to the identification of 1024 CTL, 80 HTL, and 172 LBL prioritized epitopes, which were then ranked and clustered to prefer the most immunogenicity epitopes to include in novel vaccine construct. The chosen Construct (Con2) was modified for dual-plasmid insertion, facilitating the expression of three different vaccine constructs which were predicted by C-ImmSim to stimulate robust IFN-γ/IL-2 production and enhance IgM/IgG antibody secretion. Utilizing *in silico*, next-generation vaccine design, we propose several vaccine candidates that warrant further validation through *in vitro* laboratory testing and *in vivo* studies to assess their immunological efficacy.

**Supplementary Information:**

The online version contains supplementary material available at 10.1038/s41598-026-50570-7.

## Introduction

Cystic Echinococcosis (CE) is a global zoonosis, affecting an estimated one million people and causing 183,573 disability-adjusted life years (DALYs) annually (WHO). The disease remains endemic across all countries within the Eastern Mediterranean Regional Office (EMRO) region, particularly in pastoral areas that provide favorable conditions for parasite transmission^1^. The causative agent, *Echinococcus granulosus*, is a parasitic tapeworm whose life cycle requires dogs as definitive hosts—harboring the adult intestinal stage—and livestock as intermediate hosts, where the larval (metacestode) form develops within tissues^2^.

Developing vaccines against parasitic diseases poses a considerable scientific challenge due to several factors: their intricate life cycles involving multiple hosts and developmental stages^3^ and their highly evolved mechanisms to evade or suppress host immune responses^4^. The antigenic variability and diversity of epitopes contribute significantly to this immune evasion capacity^5^.

To address these challenges, researchers have adopted advanced methodologies that integrate artificial intelligence tools to analyze large datasets and identify promising peptide sequences for therapeutic or preventive use^6^. Recent studies have increasingly focused on high-throughput analyses of parasite core proteomes to identify vaccine candidates using immunoinformatic platforms and computational tools^7–9^.

Typically, designed multi-epitope vaccines consist of Cytotoxic T-Lymphocyte (CTL), Helper T-Lymphocyte (HTL), and Linear B-Lymphocyte (LBL) epitopes connected by linkers, often combined with an adjuvant to enhance the immune response and mimic natural immunity^10^. The superfolder green fluorescent protein (sfGFP) tag, known for improving solubility, protein folding, and immunogenicity, may also serve as an adjuvant component to strengthen immune activation against the fused vaccine construct^11,12^. This study aimed to employ immunoinformatics approaches to identify and optimize protective epitopes for the development of a multi-epitope vaccine against hydatidosis, utilizing superfolder green fluorescent protein (sfGFP) as an adjuvant to improve antigen stability and immune response stimulation.

## Methodology

### Secreted core valuable proteome study

#### Core proteome compilation and redundancy reduction

The initial step involved compiling the core proteome. To accomplish this, all available protein sequences from three *Echinococcus* species (ECHGR, ECHMU, 9CEST) and nineteen isolates (see supplementary table; *Echinococcus* Isolates sheet) were collected from the UniProt database (accessed 28 September 2024) and merged into a single fasta file. Redundant sequences were then removed using CD-HIT (version 4.8.1), which clustered proteins sharing 90% sequence identity (clustering threshold = 0.9)^13^.

#### Excretory/secretory protein (ESP) identification and transmembrane filtration

The (ESPs) proteins were identified using Three signal peptide prediction tools SignalP-6^14^, TMHMM-0.2^15^, and Phobius^16^ were used for ESP prediction. Proteins predicted by SignalP-6 with a score above the 0.5 threshold were retained as candidates for classical secretion. This threshold corresponds to SignalP’s recommended discriminatory cutoff for [e.g., Gram-negative bacteria] to ensure high-confidence identification of signal peptides. Subsequently, candidate sequences were analyzed with TMHMM-2.0 and Phobius to predict transmembrane (TM) helices. To filter out integral membrane proteins, sequences predicted to contain more than two TM helices by either tool were excluded. This conservative threshold ensures the selection of soluble secreted proteins or those with minimal membrane association as mentioned in previous studies^17,18^. To eliminate unwanted sequences, TargetP^19^ was employed to remove mitochondrial signal peptides, and ScanProsite^20^ with the PS00014 pattern was used to exclude proteins containing an endoplasmic reticulum (ER) retention motif.

#### Homology, toxicity, antigenicity filtration

Potential human homologs of *Echinococcus* proteins were identified using standalone BLASTp^21^ executed in Windows PowerShell with an E-value cutoff of 1e − ^03^, after downloading all *Homo sapiens* (ID = 9606) protein sequences. ToxinPred2^22^ was utilized to differentiate between toxic and non-toxic proteins, while VaxiJen v2.0^23^ was used to assess antigenicity. Finally, the filtered protein sequences were subjected to immunogenicity analysis, and the Galaxy platform^24^ was employed for visualization and interpretation of the resulting data.

Note: All external databases and web servers used in this study were accessed between 28 September and 6 November 2024.

### Immunogenicity study

#### Epitopes prediction

The most prevalent HLA alleles in the Syrian population were used for T-cell epitope prediction using IEDB tools (accessed January-March 2025). For MHC Class I, the alleles included HLA-A01:01, HLA-A24:02, HLA-A03:01, HLA-A02:01, HLA-B51:01, and HLA-B35:01; while for MHC Class II, HLA-DRB1 11:01 and HLA-DRB1 11:04 were used^25,26^. The IEDB standalone tool was utilized to predict 9-amino acid (aa) CTL epitopes using three different methods: Consensus, NetMHCpan, and ANN. For HTL epitopes, Consensus3 (v2.22) and NetMHCIIpan (v4.1) were used to predict 12, 15, and 18-aa peptides^27^. Linear B-cell epitopes were predicted using the BepiPred-3.0 server with a threshold of 0.1512. A custom script was developed to extract these linear (12aa, 15aa, 16aa) epitopes and calculate the mean linear epitope score per protein^28^.

This analysis was conducted only on proteins containing MHC I and II ligands that show immunological relevance and belong to the ECHGR lineage. Discontinuous B-cell epitopes were predicted using the DiscoTope (v1.1a)^29^ and ElliPro (v3.0)^30^ servers enable us to calculate Discontinuous epitope score. 3D Protein structure were predicted by AlphaFold v2.0 platform (accessed 13 March 2025)^31^ and analyzed locally. DiscoTope identified epitope residues with a threshold score of ≥ − 7.7, while ElliPro recognized residues with scores above 0.5. Custom scripts were employed to quantify the number of epitope residues exceeding these thresholds for each protein, and to compute their corresponding scores using both DiscoTope and ElliPro, as outlined above.

### Epitopes evaluation

1. Immunogenicity: Six MHC I alleles were analyzed using the IEDB Class I Immunogenicity tool to identify 9-mer peptide binders with an immunogenicity threshold of 0.1. Additionally, CD4 T-cell Immunogenicity analysis was performed to detect 15-mer MHC II immunogenic binders^27^.
2. Cytokine Inducers: The updated IFNepitope2 tool (accessed 7 March 2025)^32^ was employed to identify IFN-γ–inducing peptides among both MHC I and MHC II binders. The IL4pred web server (accessed 4 February 2025)^33^ was used to predict IL-4–inducing MHC II epitopes, while IL-10Pred (accessed 7 February 2025)^34^ was applied to exclude MHC II epitopes capable of inducing IL-10 responses.
3. Immunoglobulin Prediction: B-cell epitopes were analyzed using the IgPred web server (accessed 24 March 2025) to predict their ability to induce specific antibody classes (IgG, IgE, and IgA)^35^, with a prediction threshold set at 0.9.
4. Population coverage: Analysis for the selected epitopes was performed with the IEDB population coverage computation tool.

Note: For the Immunogenicity study, locally installed tools, the download and installation dates correspond to the same period.

### Vaccine epitopes normalization

To identify the most effective CTL, HTL, and LBL epitopes, a normalization process was performed to assign a score to each epitope individually. This score was calculated by summing the normalized values across all evaluation criteria used, as detailed below:

$$\begin{aligned} {\mathrm{CTL}}\,{\mathrm{epitope}}\,{\mathrm{score}} & = {\mathrm{Antigenicity}}\,{\mathrm{score}} + {\mathrm{Immunogenicity}}\,{\mathrm{score}}\left( {{\mathrm{CD8}}} \right) \\ & \quad + {\mathrm{MHCI}}\,{\mathrm{prediction}}\,{\mathrm{frequency}} \\ & \quad + {\mathrm{MHCI}}\,{\mathrm{allele}}\,{\mathrm{Count}}\left( {{\mathrm{Promiscuous}}} \right) \\ \end{aligned}$$

$$\begin{aligned} {\mathrm{HTL}}\,{\mathrm{epitope}}\,{\mathrm{score}} & = {\mathrm{Antigenicity}}\,{\mathrm{score}} + {\mathrm{Immunogenicity}}\,{\mathrm{score}}\left( {{\mathrm{CD4}}} \right) \\ & \quad + {\mathrm{MHCII}}\,{\mathrm{prediction}}\,{\mathrm{frequency}} \\ & \quad + {\mathrm{MHCII}}\,{\mathrm{core}}\,{\mathrm{repeat}}\,{\mathrm{count}} \\ \end{aligned}$$

$${\mathrm{LBL}}\,{\mathrm{epitope}}\,{\mathrm{score}} = {\mathrm{Antigenicity}}\,{\mathrm{score}} + {\mathrm{Immunogenicity}}\,{\mathrm{score}}\left( {{\mathrm{Ig}}} \right) + {\mathrm{B}}\,{\mathrm{Cell}}\,{\mathrm{protein}}\,{\mathrm{score}}$$

While B Cell protein score was calculated as follows:

$$\begin{aligned} {\mathrm{B}} - {\mathrm{Cell}} - {\mathrm{protein}}\,{\mathrm{score}} & = {\mathrm{Linear}}\,{\mathrm{epitope}}\,{\mathrm{score}} + {\mathrm{Discontinuous}}\,{\mathrm{epitope}}\,{\mathrm{score}} \\ & = {\mathrm{Average}}\,{\mathrm{count}}\,{12},{15},{16}\,{\mathrm{aa}}\left( {{\mathrm{BepiPred}}} \right) \\ & \quad + {\mathrm{Average}}\,{\mathrm{linear}}\,{\mathrm{score}}\,{12},{15},{16}\,{\mathrm{aa}} \left( {{\mathrm{BepiPred}}} \right)\\ & \quad + {\mathrm{Dis}} - {\mathrm{Epitopes}}\,{\mathrm{count}}\left( {{\mathrm{ElliPro}}} \right) \\ & \quad + {\mathrm{Dis}} - {\mathrm{Epitopes}}\,{\mathrm{Score}}\left( {{\mathrm{ElliPro}}} \right) \\ & \quad + {\mathrm{Dis}} - {\mathrm{Epitopes}}\,{\mathrm{count}}\left( {{\mathrm{DiscoTope}}} \right) \\ \end{aligned}$$

In the next step, to eliminate closely related or redundant epitopes, the Epitope Cluster tool^36^ was used to group similar epitopes into organized clusters. From each cluster, the epitope with the highest score was selected as the representative.

Linux-based software was employed to run various immunoinformatic tools, while custom Python scripts developed in the PyCharm environment^37^ were used to filter and refine the final set of accepted epitopes.

### Vaccine construction and structure validation

Epitopes were ranked based on their final scores, and the top-performing candidates were selected for inclusion in the vaccine construct. Appropriate linkers and an adjuvant were incorporated to enhance immunogenicity and structural integrity.

We employed a range of tools to evaluate proposed construct sequences and select the most suitable one based on physicochemical properties and solubility. This included analysis using ProtParam^38^ and Protein-Sol^39^ as well as immune response simulation with C-ImmSim, focusing on selected HLA alleles (A01:01, A02:01, B35:01, B51:01, DRB111:01, and DRB111:04)^40^. TMHMM-2.0 was utilized to identify and exclude epitopes located within transmembrane helices. Secondary structure prediction was performed using PSIPRED^41^ while AlphaFold2^42^ was used for 3D structure prediction leveraging MMseqs2 for construct generation and selecting optimal models based on predicted local-distance difference test (pLDDT) and predicted template modeling (pTM) scores. Structural validation was conducted via the MolProbity server^43^ which assessed favored residues and Ramachandran plot Z-scores before and after refinement. The final model refinement was carried out using Galaxy Refine^44^ to ensure optimal structural geometry.

Note: Tools for Paragraph 5 utilized external databases and web servers accessed at different time between 2 April and 23 August 2025 throughout the project.

### Codon optimization and *In Silico* cloning

The EMBOSS BACKTRANSEQ tool (accessed 11 July 2025)^45^ was used to reverse-translate amino acid sequences into nucleotide sequences using *Escherichia coli* codon preferences. Subsequently, the GenSmart tool (accessed 15 July 2025)^46^ optimized the codons to enhance gene expression in both *E. coli* and human systems. The optimized gene sequences were then cloned into two different vectors, pRSET-a and pRSET-sfGFP, using Geneious software. Three restriction enzyme recognition sites were incorporated into the construct: *BamHI* at the 5′ end and *EcoRI* at the 3′ end, with *HindIII* positioned upstream of the EAAAK linker and the following Adj sequence at the 3′ end. To assess the structural stability and solubility of mRNA, RNAfold web server (accessed 5 August 2025)^47^ was employed to predict the 2D structures of the DNA transcript sequences, focusing on centroid analysis for more accurate evaluation.

## Results

### Proteins selection: *In silico*

Using CD-HIT, the initial UniProt sequence dataset was clustered into 18,528 groups, each represented by a single, non-redundant protein sequence. Signal peptide (SP) prediction tools reduced the dataset to 2603 sequences after the first filtration, as processed by a Python-based algorithm. A second filtration step refined the dataset further to 2251 protein sequences by removing seven sequences containing endoplasmic reticulum (ER) signals, 79 predicted to carry mitochondrial targeting peptides, and 266 identified as homologous to human proteins.

**Fig. 1:**
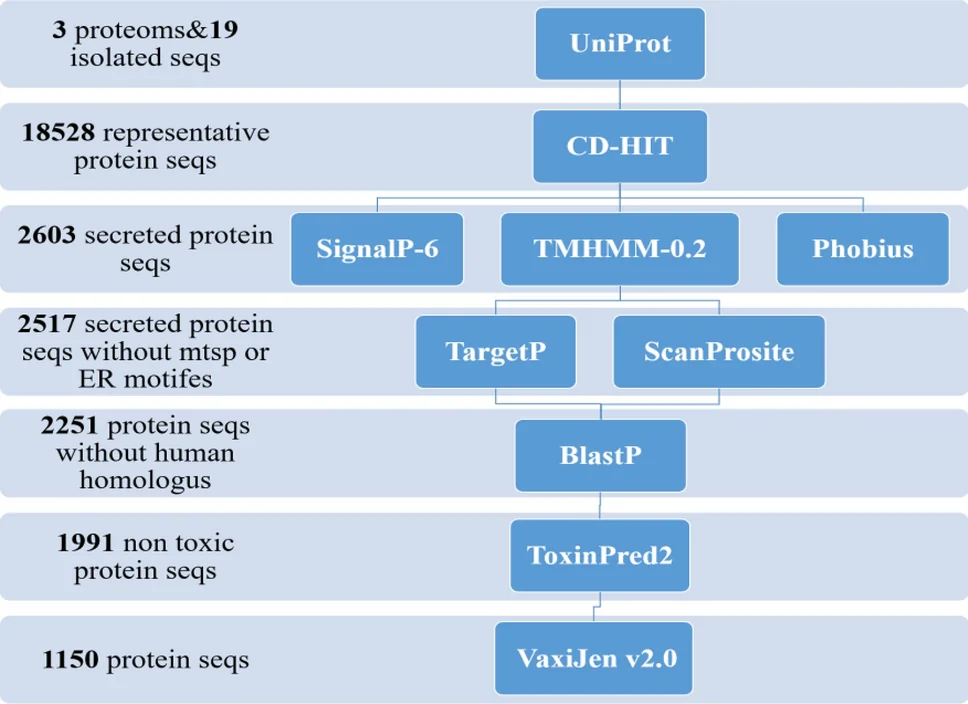
A schematic of pipeline processes for high throughput in silico protein selection.

In the third filtration stage, sequences that were toxic or lacked antigenicity were excluded. Out of the remaining 1991 proteins, only 1150 surpassed the antigenicity threshold (≥ 0.5) and were retained for subsequent immunogenicity analysis (Fig. 1).

### Epitopes selection

For CTL epitope prediction, 11,413 peptides (with ranks ≤ 2) were commonly identified by all three prediction tools and were considered for further analysis. From these, 3243 peptides were identified as immunogenic. Within this group, 2474 peptides were predicted to induce IFN-γ at a threshold of 0.49. A subset of 1129 CTL epitopes was both non-toxic and antigenic, mapping to 390 distinct proteins (see supplementary table; MHCI Epitopes sheet). Similarly, HTL epitope prediction yielded 12,817 core shared representative 15-aa peptides binding to MHC class II molecules, of which 10,429 had an immunogenicity score above 15. Among these, 713 were predicted to induce IFN-γ and IL-4 without inducing IL-10. After filtering non-toxic and antigenic properties, 126 HTL epitopes remained, corresponding to 38 unique proteins (see supplementary table; MHCII Epitopes sheet).

The previously selected MHC-evaluated proteins comprising 400 non-redundant ECHGR-matched sequences were exclusively used for B-cell epitope prediction. Linear B-cell epitopes of three lengths (12, 15, and 16 amino acids) were predicted using the BepiPred tool, yielding 19,492, 17,996, and 17,505 sequences, respectively. Among these, only 296 (12aa), 818 (15aa), and 1017 (16aa) epitopes were predicted to effectively stimulate B cells for immunoglobulin secretion. Further filtering for non-toxic and antigenic properties resulted in 271, 655, and 672 linear B-cell (LB) epitopes for each respective length. Additionally, 365 protein structures (PDB files) were assessed for discontinuous (conformational) B-cell epitopes. Only 68 proteins met the evaluation criteria across all three conformational prediction methods and were ranked accordingly (see supplementary table; B-Cell protein score sheet). Based on IEDB data^7^, the 12-aa epitope length being the most frequently observed was selected as the representative length for analysis, resulting in 172 unique linear B-cell epitopes (LBL) (see supplementary table; LB Epitopes sheet).

**Fig. 2:**
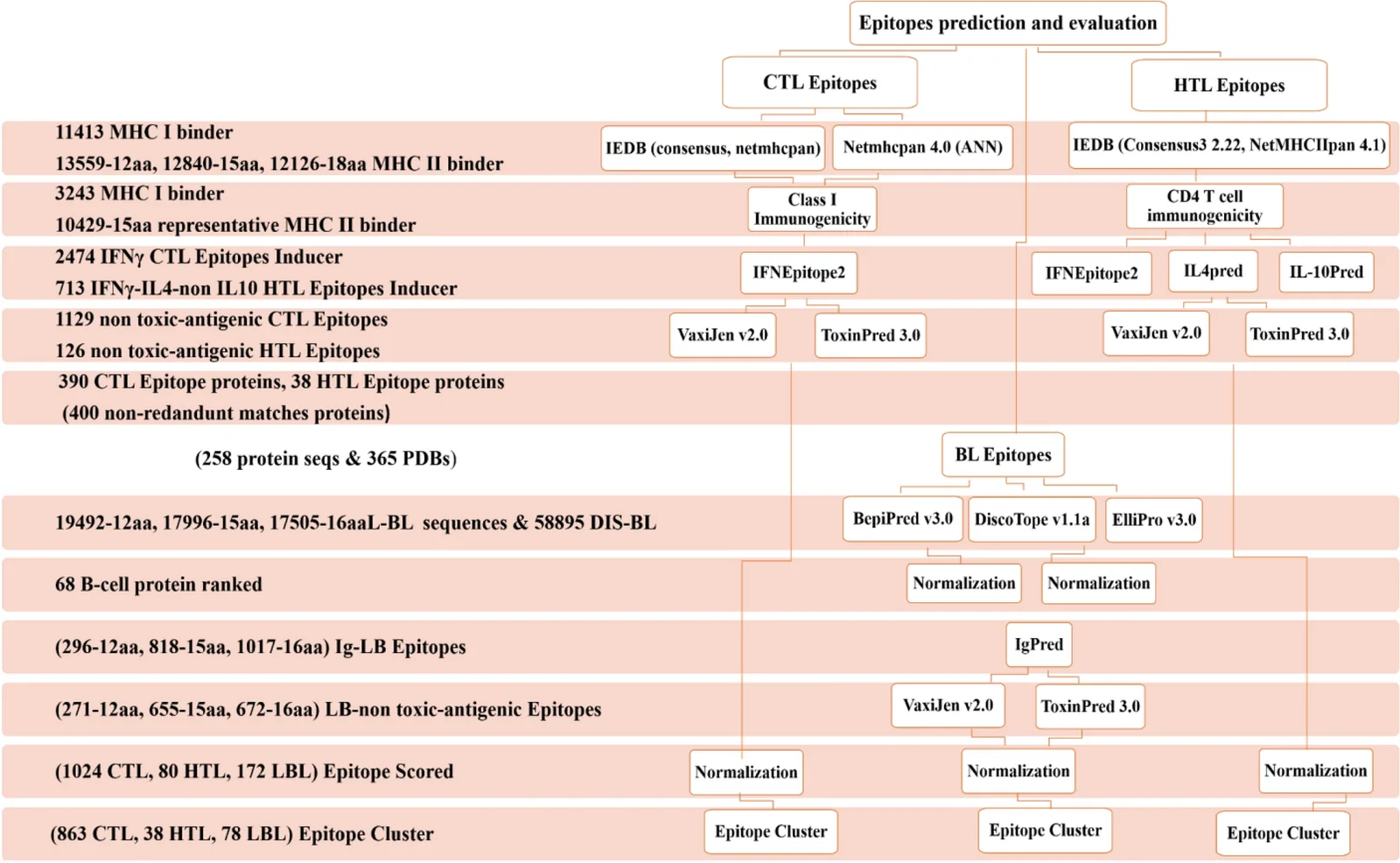
A schematic of pipeline processes for in silico epitopes prediction and evaluation.

In total, the number of immunologically validated epitopes included 1024 CTL, 80 HTL, and 172 LBL epitopes, as detailed in the supplementary table. These were further grouped into epitope clusters, resulting in 863 CTL, 38 HTL, and 78 LBL non-redundant epitope clusters (Fig. 2).

Following epitope ranking based on immunological scoring, the top candidates selected for inclusion in the vaccine formulation are listed in (Table 1).

**Table 1:** The scored epitopes Selected of preferred construct (Con2).

ECHGR protein ID	Lymphocyte	Epitope	Antigenicity score	Immunogenicity score	Cluster No	Epitope score
A0A068WWA7_ECHGR Expressed protein A0A068X2Q3_ GP46-like surface antigen	CTL	MAIPIWFRI YANWIVVGV	1.8736 0 0.7281	0.475523333 0.438433333	S 106.1 S 147.1	2.1555 1.816
W6UN40_ EG95 W6UN40_ EG95 W6UK15_ Transcription elongation factor B polypeptide 2	HTL	NNTYNFTLCIRLHSD VDIEEKLLLLSKMRK KEDKRTMKFLVRTVL	1.332 0.6377 1.1872	85.6433 93.4911 67.8421	C 10.1 C 9.1 S 25.1	1.682 1.47 1.257
A0A068WTR6_ Secreted protein C1KBK3_ Antigen B2 subunit Fragment	LBL	ALFLIFYPVKIS TAICQKLQLKIR	1.4778 1.5411	0.965 (IgE Epitope) 1.054 (IgA Epitope)	S 43.1 S 57.1	2.195 2.076

### Multi epitopes vaccine construction

**Fig. 3:**
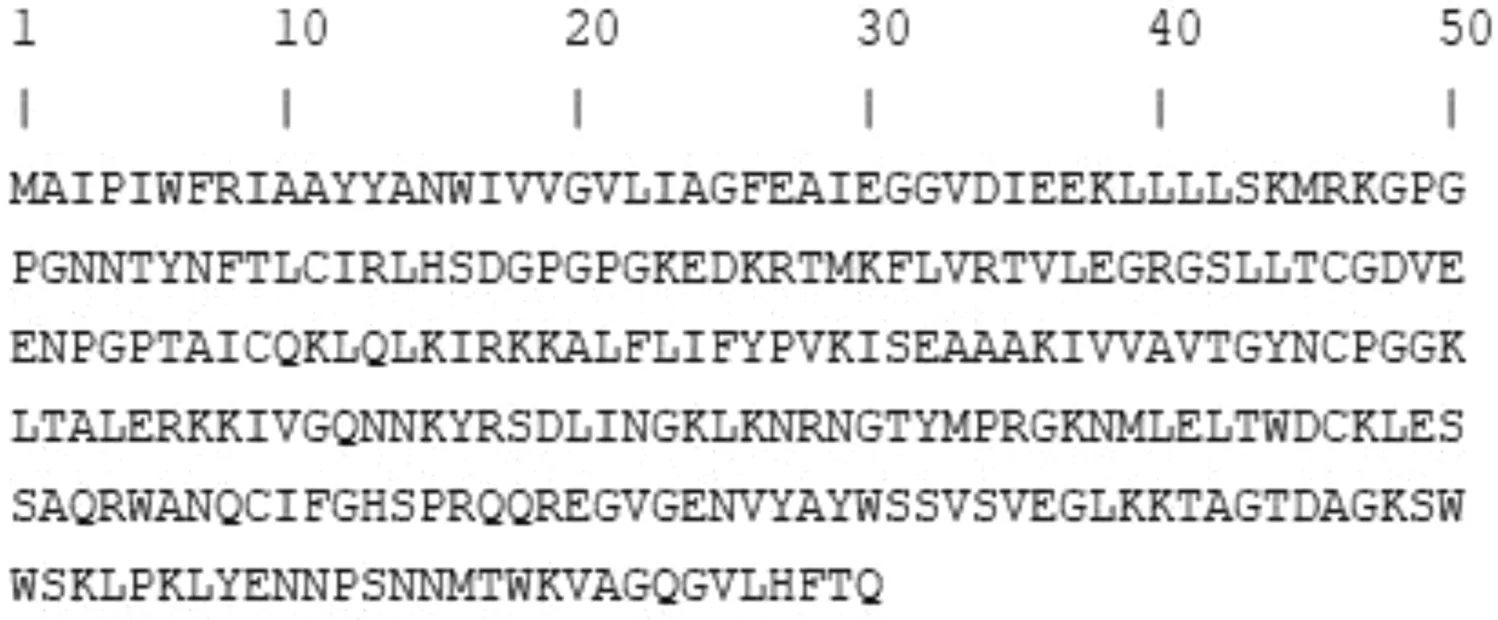
The alignment amino acid sequence of Construct 2; Multi Epitope Vaccine- rov-ASP-1 adjuvant (MEV-Adj).

The preferred construction, referred to as Construct 2 (Con2; Fig. 3), comprises seven epitopes 2 CTL, 3 HTL, and 2 LBL (Multi Epitope Vaccine: MEV) linked together using five different flexible linkers. While stable one (EAAAK) was used to bind MEV with the RovA-derived adjuvant (rov-ASP-1), known to act as a strong TLR2/4 agonist, thereby promoting a balanced Th1/Th2 immune response (Table 2)^48^. For the Southwest Asian population, the projected immunogenic coverage of construct 2 reached 58.02%, based on combined MHC class I and II epitope binding predictions.

**Table 2:** The linkers and adjuvant included in multi-epitope vaccine.

Linker	Sites
*AAY	CTL Epitopes
*LIAGFEAIEGG	CTL—HTL Epitopes (from tetanus toxin)
*GPGPG	HTL Epitopes
*EGRGSLLTCGDVEENPGP	HTL -LBL Epitopes (T2A)
*KK	LBL Epitopes
**EAAAK	Vaccine- Adjuvant
IVVAVTGYNCPGGKLTALERKKIVGQNNKYRSDLINGKLKNRNGTYMPRGKNMLELTWDCKLESSAQRWANQCIFGHSPRQQREGVGENVYAYWSSVSVEGLKKTAGTDAGKSWWSKLPKLYENNPSNNMTWKVAGQGVLHFTQ	Adjuvant (rov-ASP-1 AAB69625.2)

*Flexible linker, ** stable linker.

Construct 2 (Con2) consisted of 280 amino acids with a molecular weight of 31,171.14 Da. It was predicted to be stable (instability index = 21.16), hydrophilic (GRAVY score = − 0.333), antigenic (antigenicity score = 0.7847), soluble (solubility = 0.553), and non-allergenic. Codon optimization using the GenSmart tool confirmed that the corresponding nucleotide sequence had an acceptable GC content of 51.07% and Codon Adaptation Index (CAI) is 0.663.

### Vaccine structures cloning and transcripts validation: *In Silico*

**Fig. 4:**
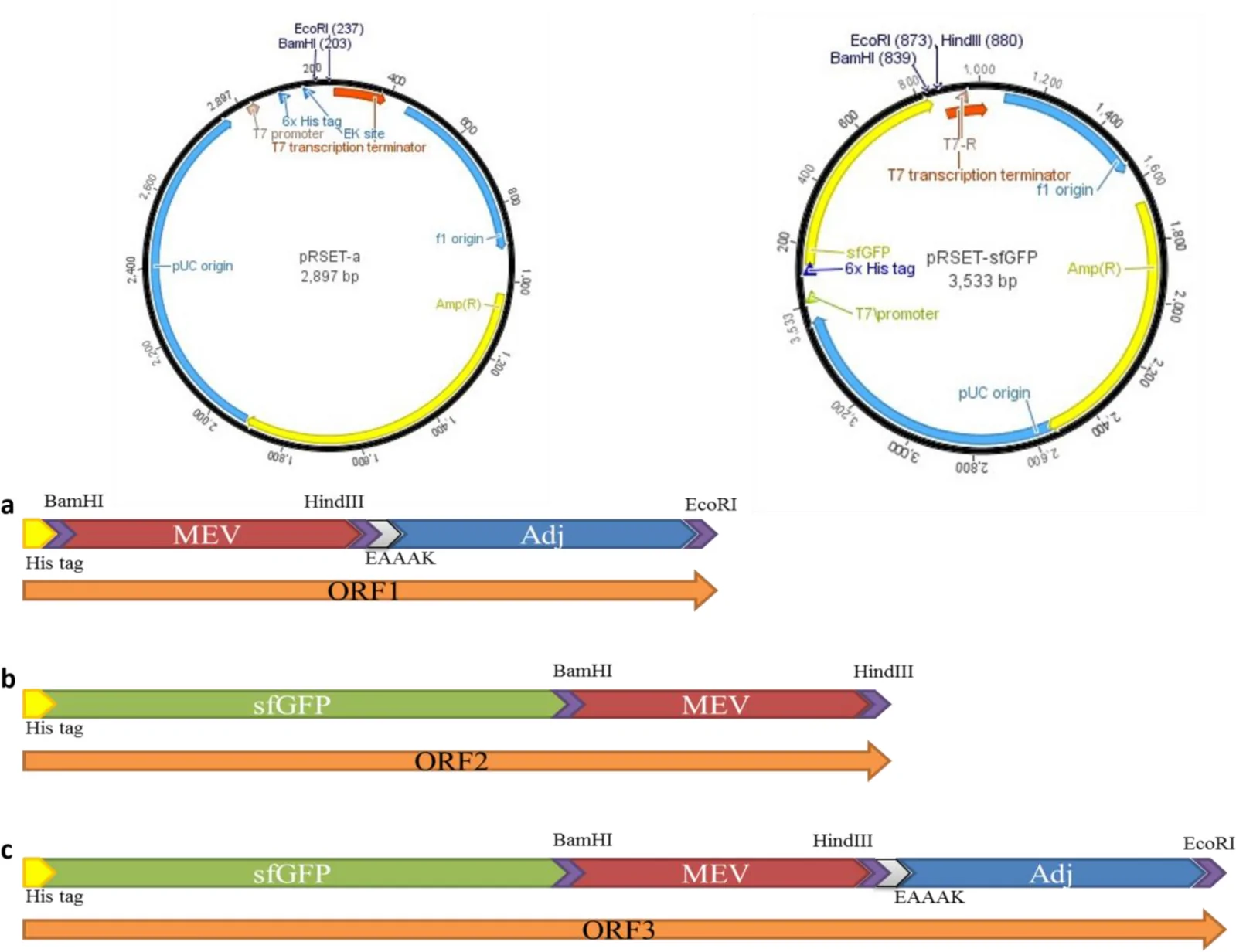
In silico cloning of three vaccine’s structures: (**a**) ORF of Con2 (MEV-Adj) was cloned into pRSET-a plasmid. (**b**) ORF of MEV fusion into sfGFP was cloned into pRSET-sfGFP plasmid. (**c**) ORF of Con2 fusion into sfGFP was cloned into pRSET-sfGFP plasmid.

Three different open reading frames (ORFs) were generated from the modified insert sequence following cloning into vectors (Fig. 4):

1. MEV-Adj was digested with *BamHI* and *EcoRI* and cloned into the pRSET-a plasmid.
2. sfGFP-MEV was digested with *BamHI* and *HindIII* and cloned into the pRSET-sfGFP plasmid.
3. sfGFP-MEV-Adj was digested with *BamHI* and *EcoRI* and cloned into the pRSET-sfGFP plasmid.

**Fig. 5:**
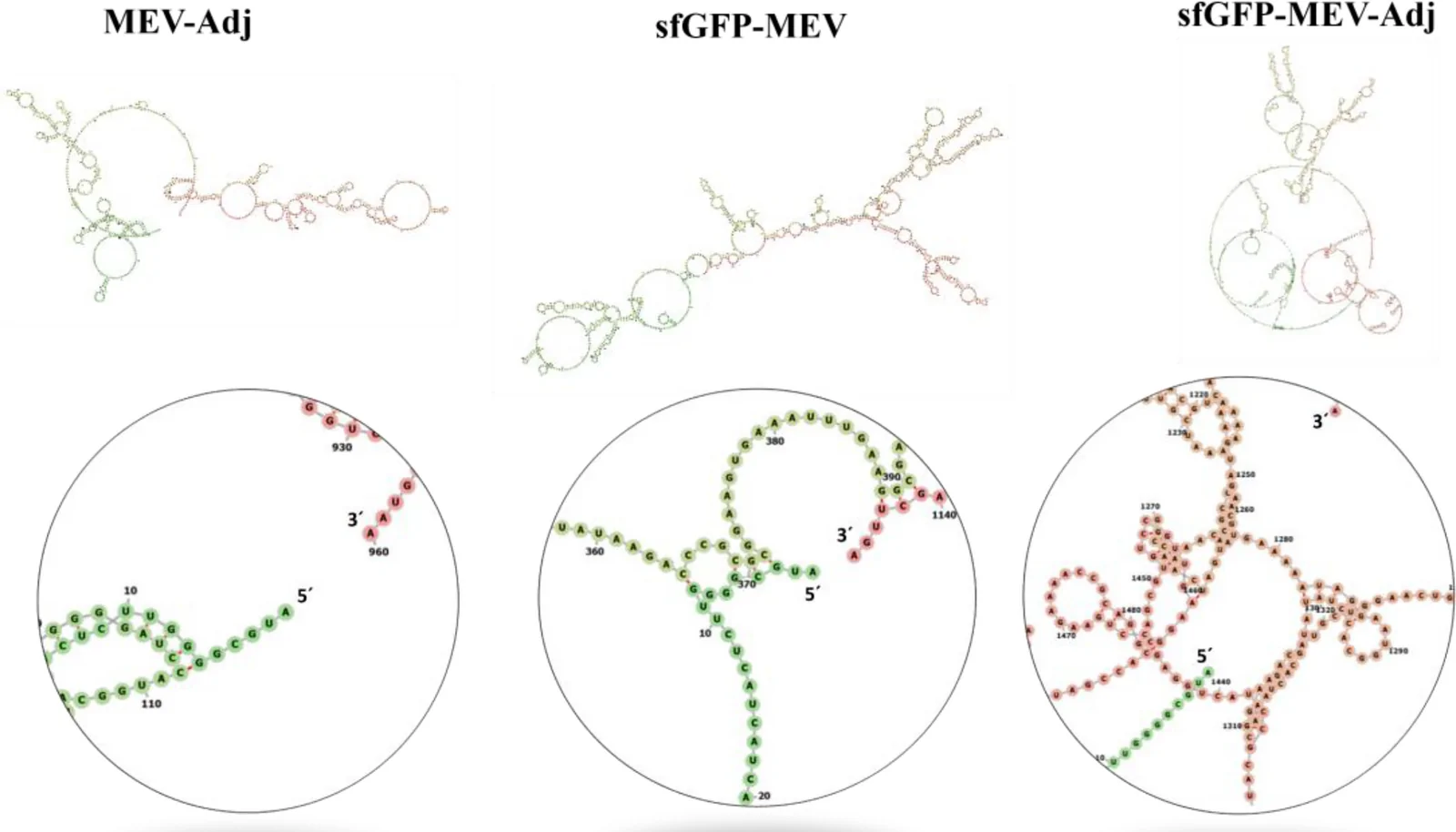
The secondary predicted structure of mRNA for three vaccine’s structures. There are no pseudoknots or hairpins at the 5’ end of the projected mRNA structure.

Additionally, the secondary structures of the messenger RNA sequences, predicted by the RNA fold server, demonstrated thermodynamic stability with minimum free energy (MFE) values of − 227.61, − 316.10, and − 350.10 kcal/mol for ORFs 1, 2, and 3, respectively. Moreover, analysis of the first 20 nucleotides revealed no pseudoknots or long, stable hairpin formations in any of the three mRNA structures, as shown by the thermodynamic data (Fig. 5).

### Vaccines structures validation: *In Silico*

Protein analysis tools were used to compare the three final amino acid vaccine constructs (319, 381, and 531), (Table 3).

**Table 3:** Parameters used for evaluation of the three vaccine’s structures.

Tool	Parameter	MEV-Adj	sfGFP-MEV	sfGFP-MEV-Adj
ProtParam	No of amino acids: Theoretical pI: Molecular weight: Formula: Estimated half-life (Escherichia coli, in vivo) Instability index (II) Aliphatic index: (GRAVY):	319 9.46 35,613.92 Da C_1583_H_2491_N_453_O_452_S_16_ > 10 h 21.70 76.74 − 0.471	381 7.73 42,465.60 Da C_1903_H_2979_N_523_O_552_S_14_ > 10 h 26.23 82.86 − 0.352	531 9.07 59,212.67 Da C_2644_H_4143_N_739_O_768_S_20_ > 10 h 23.03 78.74 − 0.444
Protein-Sol	Predicted solubility: pI:	0.458 10.060	0.313 8.650	0.327 9.630
PSIPRED	α-helix β-strand Random coils	11 (40.74%) 2 (7.41%) 14 (51.85%)	11 (20%) 17 (30.9%) 27 (49.1%)	12 (21.05%) 16 (28.07%) 29 (50.88%)
VaxiJen v2.0	Antigenicity	0.7814	0.9102	0.8274
MolProbity Ramachandran Plot Z score	Favored residues Allowed residues Whole: Helix: Sheet: Loop:	98.4% (312/317) 99.7% (316/317) 0.93 (0.41), residues: 317 0.92 (0.33), residues: 156 1.72 (1.26), residues: 16 0.31 (0.50), residues: 145	98.7% (374/379) 99.7% (378/379) 0.78 (0.38), residues: 379 − 1.26 (1.21), residues: 18 0.92 (0.35), residues: 161 0.48 (0.40), residues: 200	98.5% (521/529) 99.8% (528/529) 1.04 (0.34), residues: 529 0.59 (0.42), residues: 110 0.96 (0.35), residues: 165 0.73 (0.39), residues: 254
Ellipro	No of Predicted Linear Epitopes No of Predicted Discontinuous Epitopes	9 13	15 11	15 11
C-IMMSIM No of Predicted	B-cell Epitopes (MHCI Epitopes; non-binding event score) A01:01 A 02:01 B 35:01 B 51:01 (MHCII Epitopes; non-binding event score) DRB1 11:01 DRB1 11:04	9 (0; 1.000000) (2; 0.945718) (1; 0.976095) (5; 0.861428) (20; 0.432298) (15; 0.427643)	9 (2; 0.979918) (5; 0.907997) (1; 0.997075) (7; 0.784959) (19; 0.472435) (13; 0.514521)	14 (1; 0.979918) (5; 0.907997) (2; 0.973308) (9; 0.743264) (26; 0.379661) (15; 0.397415)

**Fig. 6:**
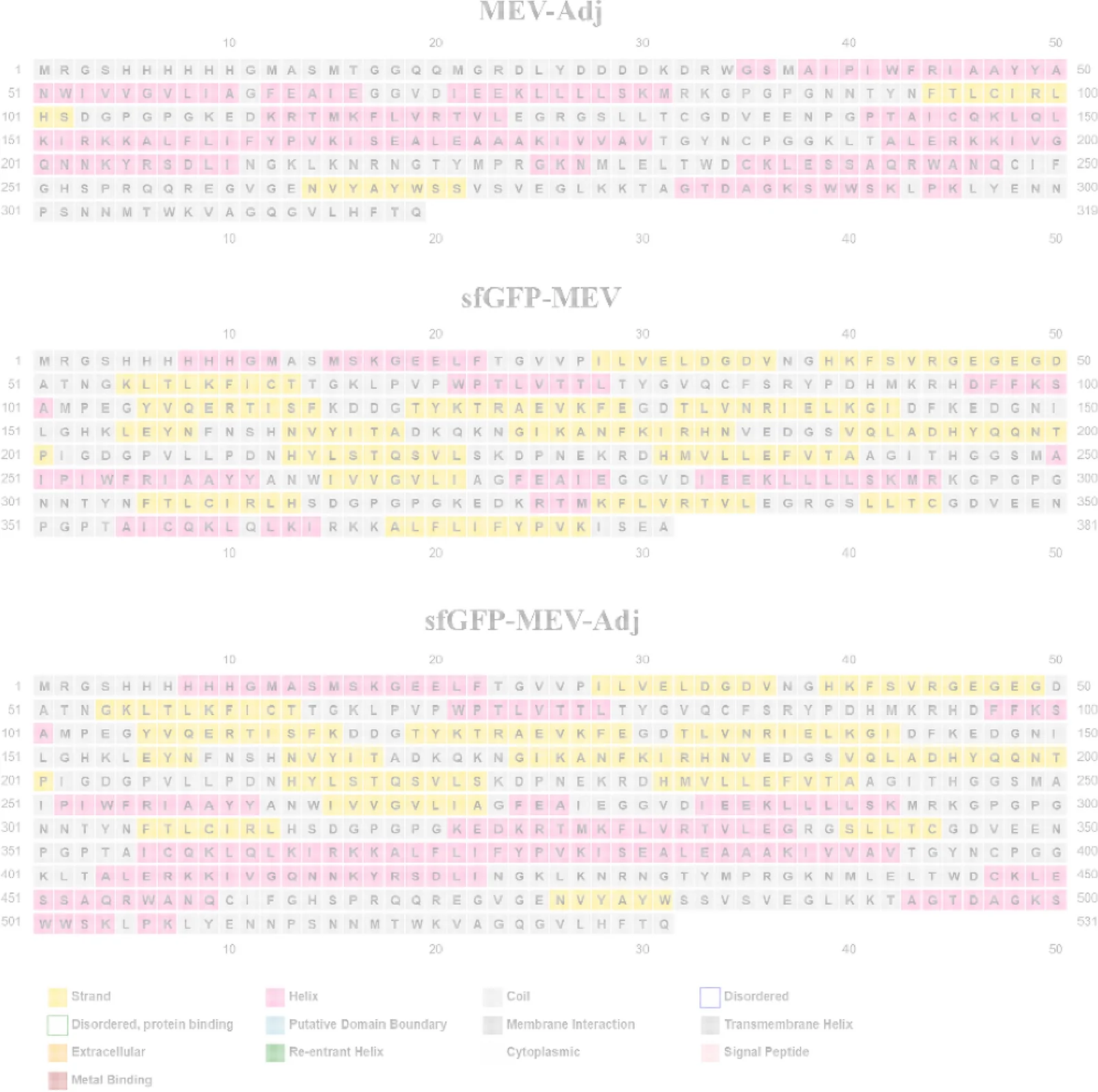
Secondary structure prediction of vaccine constructs. The secondary structure of the three designed vaccine constructs (MEV-Adj, sfGFP-MEV, sfGFP-MEV-Adj) was predicted from their amino acid sequences using PSIPRED.

All of them exhibited favorable physicochemical characteristics with antigenicity scores above 0.5. However, among these, only the MEV-Adj vaccine structure was predicted to have higher solubility than the others. The PSIPRED server predicted diverse secondary (2D) structural conformations for these constructs (Fig. 6).

Using AlphaFold2 to predict 3D structure, we selected top-ranked models for three constructs (MEV-Adj, sfGFP-MEV, and sfGFP-MEV-Adj) with pLDDT scores of (61.2, 75.9, 72.1) and pTM scores of (0.516, 0.507, 0.632), respectively. From five additional models generated by Galaxy Refine, we then selected the final optimal model based on its superior predicted RMSD (pRMSD) values: 0.522 Å (MEV-Adj), 0.445 Å (sfGFP-MEV), and 0.457 Å (sfGFP-MEV-Adj).

**Fig. 7:**
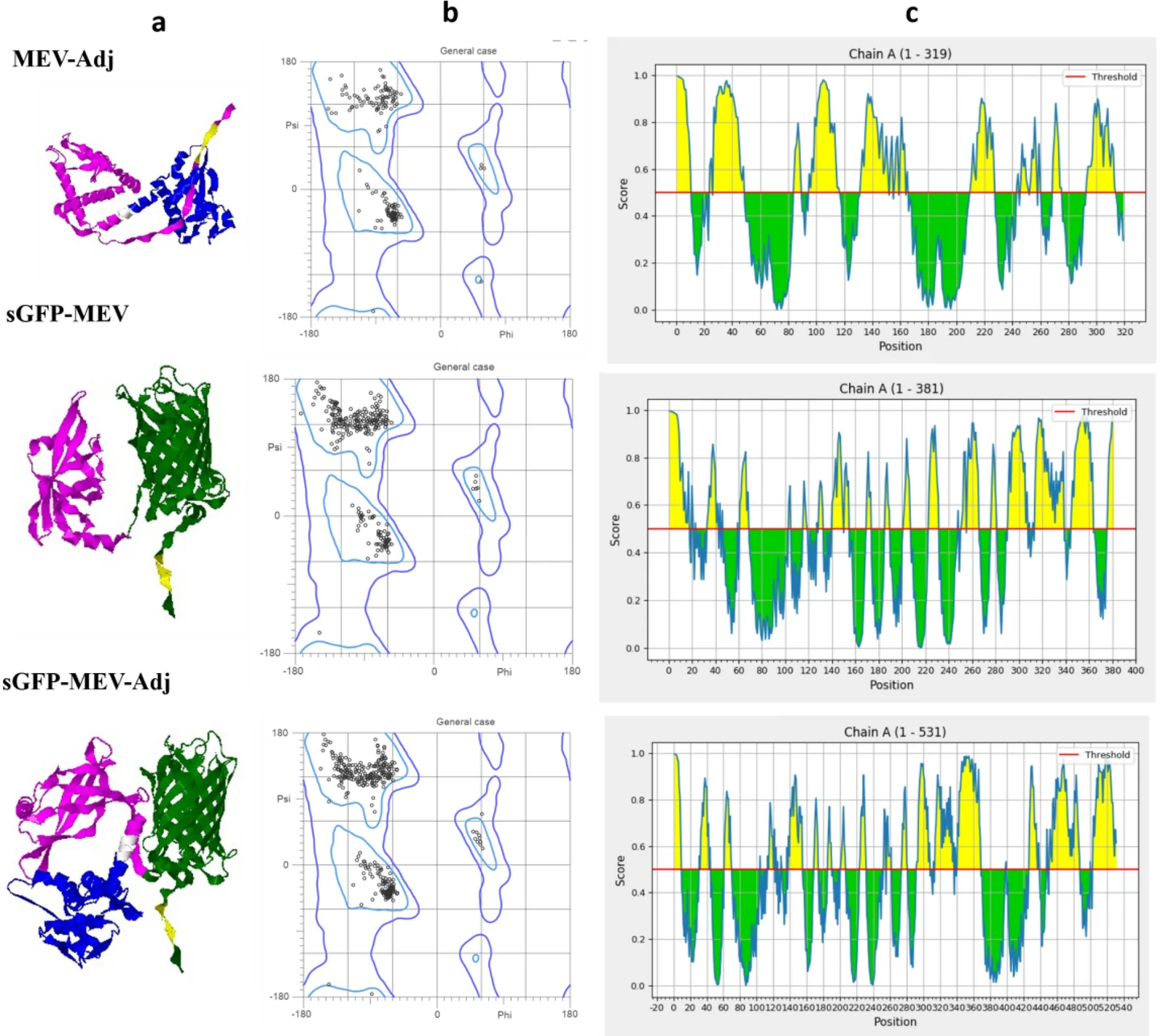
Comparison between the three vaccine’s structures: (**a**) 3D predicted structure by AlphaFold2. (**b**) Ramachandran plot by MolProbity. (**c**) 2D score chart for residue vaccine’s structures by ElliPro above threshold = 0.5

MolProbity analysis indicated that the refined 3D (Fig. 7, a) vaccine models exhibited good structural quality, with over 98% of residues located within favored and allowed regions (Fig. 7, b). The overall |Rama Z| scores for the three structures (MEV-Adj, sfGFP-MEV, and sfGFP-MEV-Adj) were 0.93, 0.78, and 1.04, respectively, all of which were below 2, indicating that the models exhibit proper stereochemical geometry. Additionally, evaluation of the PDB files using ElliPro (with a 0.5 score threshold and 6 Å distance) revealed an enrichment of both linear and discontinuous epitopes across the three structures, with many residues exceeding the minimum score (Fig. 7, c).

**Fig. 8:**
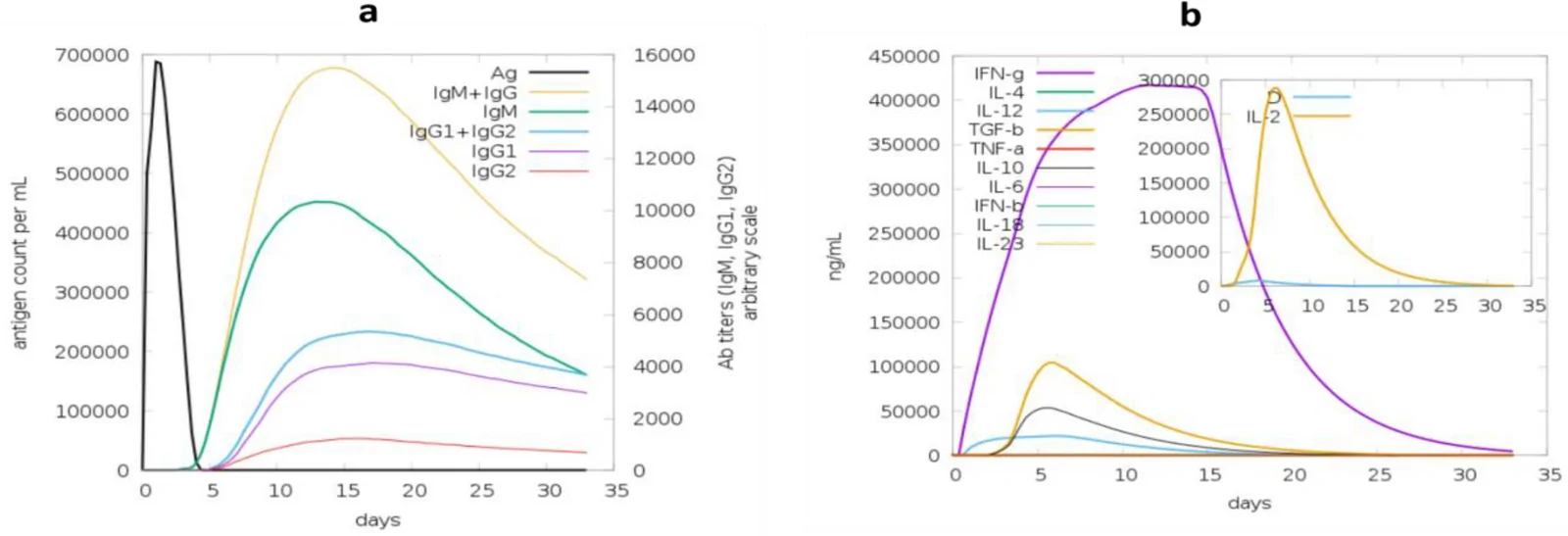
Immune simulation by C-ImmSim of sfGFP-MEV-Adj. (**a**) Increase of antibody response indicates primarily Th2 response. (**b**) A high level of IFN-γ and IL-2 suggest a strong Th1 response.

C-ImmSim analysis demonstrated the vaccine’s potential to elicit a strong adaptive immune response, as modeled by the activation of T- and B-cell memory populations and elevated predicted secretion of IFN-γ and IL-2. The simulation also indicated a robust humoral response, evidenced by increased simulated levels of immunoglobulins, including IgG, IgM, and their isotypes. (Fig. 8).

## Discussion

Excretory/secretory (ES) proteins play a pivotal role in numerous biological processes, particularly in mediating host–parasite interactions. Their unique structural and functional properties make them promising targets for therapeutic, diagnostic, and immunological applications^17,18,49^. A recent study characterized the ES proteins of *E. multilocularis* highlighting their potential as novel targets for intervention strategies^17^. In this work, we focused on identifying ES proteins within the *Echinococcus* core proteome that display key characteristics: lack of homology with human proteins to avoid autoimmunity, strong antigenicity, and non-toxicity. The subsequent objective was to validate and rank these proteins based on their immunogenic potential to facilitate the design of effective multi-epitope vaccine. Successful vaccines elicit robust and balanced immune responses; therefore, our design aimed to stimulate B cells, TH, and CT lymphocytes. Although CTLs may play a secondary role, maintaining an appropriate Th1/Th2 balance and enhancing antibody-dependent cellular cytotoxicity (ADCC) are critical for achieving long-lasting protective immunity^50,51^.

Each predicted epitope—restricted by MHC class I and class II molecules—was thoroughly evaluated and ranked according to antigenicity, immunogenicity, prediction frequency, and allele promiscuity (for MHC I), while core repeat motifs were considered for MHC II epitopes. Linear B-cell epitopes were also prioritized based on calculated B-cell protein scores. This ranking methodology, proven effective in a previous vaccine discovery study against *Toxoplasma gondii*^7^, ensured a systematic and evidence-driven selection of high-value candidates.

Notably, several prioritized epitopes in our study corresponded to proteins previously proposed as vaccine targets against echinococcosis such as EG95 and the Antigen B2 subunit fragment^52,53^ and leishmaniasis (gp46)^54^. This convergence reinforces the reliability of our bioinformatic filtering pipeline and validates the biological relevance of our selected protein candidates.

Using two cloning vectors, we successfully generated three distinct vaccine constructs following Con2 modulation with restriction enzymes. Among these, the two sfGFP-fusion constructs exhibited higher antigenicity and contained more accessible B-cell epitopes, albeit with slightly reduced solubility compared to the third construct, as indicated by Protein-Sol tool analysis. This result aligns with previous findings suggesting that sfGFP, despite its solubility-enhancing potential, can occasionally compromise overall fusion protein solubility^55,56^.

Simulation results obtained from the C-ImmSim platform, which predicts immune responses based on amino acid sequences (Table 3), offered further valuable insights. The sfGFP tag exhibited high immunogenicity, containing several strong MHC I epitopes, in agreement with previous studies^11,55^. Similarly, the MEV-Adj construct showed strong MHC II epitope presentation, aligning with earlier findings on rOv-ASP-1—a helminth-derived adjuvant demonstrated to enhance both Th1- and Th2-type immune responses and to surpass conventional Adjuvants in stimulating antibody production and cytokine activity^48^. Among the evaluated models, the sfGFP-MEV-Adj hybrid displayed the most favorable overall immunological profile, characterized by the lowest non-binding event probability (~ 0.38), the highest diversity of MHC II epitopes, and a broad spectrum of MHC I epitopes originating from both the sfGFP and Con2-Adj regions (e.g., A0201 and B5101 binders). Additionally, it contained the greatest number of predicted linear epitopes according to the Parker B-cell scale^57^, indicating strong potential for broad and effective immune activation.

## Conclusion

This study successfully identified and characterized key excretory/secretory proteins from the *Echinococcus* core proteome with high antigenic potential and minimal risk of autoimmunity or toxicity. Through an integrated immunoinformatic and AI-based pipeline, we predicted, filtered, and prioritized epitopes suitable for multi-epitope vaccine design. Among the constructed vaccine models, the sfGFP-MEV-Adj fusion exhibited the most promising immunogenic profile, with broad MHC binding capacity and strong B- and T-cell activation potential. These findings provide a solid foundation for future experimental validation and the development of an effective vaccine against cystic echinococcosis.

## Supplementary Information

Supplementary Information.

## Data Availability

All obtained results during the current study are included in this article and are available from the corresponding author upon reasonable request.
